# Uranium standards in drinking water: An examination from scientific and socio-economic standpoints of India

**DOI:** 10.1007/s11356-024-34352-0

**Published:** 2024-07-15

**Authors:** Sanjay K. Jha, Aditi C. Patra, Gopal P. Verma, Vivekanand Jha, Dinesh K. Aswal

**Affiliations:** 1 Health Physics Division, Health Safety & Environment Group, BARC, Mumbai, 400085 India; 2 Health Safety & Environment Group, BARC, Mumbai, 400085 India; 3 Homi Bhabha National Institute, Anushaktinagar Mumbai, 400094 India

**Keywords:** Uranium, Drinking water, Guideline values, Toxicity, Environment

## Abstract

The detection of uranium in drinking water has ignited concerns among the public, regulators, and policymakers, particularly as around 1% of the 55,554 water samples in India have shown uranium levels surpassing the 60 µg/l guideline established by the Atomic Energy Regulatory Board (AERB) based on radiological toxicity. Further, the Bureau of Indian Standard (BIS), has given a limit of 30 µg/l, which is derived from World Health Organization (WHO) guidelines. Besides the chemical and radiological aspects associated with uranium, factors such as technological constraints in water purification, waste management, environmental factors, and socio-economic conditions significantly influence these guideline values, which are often overlooked. This manuscript explores the variations in approaches for establishing guideline values and highlights the uncertainties arising from dependence on various variables such as intake and usage patterns, inter- and intra-species distinctions, and epidemiological data. A critical analysis indicates that adherence to global guidelines may result in some undesirable environmental issues. By considering factors such as population dynamics, socio-economic conditions, and geological influences, we suggest that limit of 60 µg/l for uranium in drinking water is appropriate for India.

**Graphical abstract:**

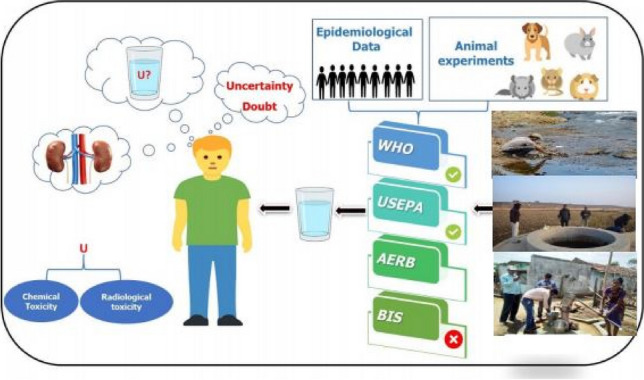

## Introduction

**Fig. 1:**
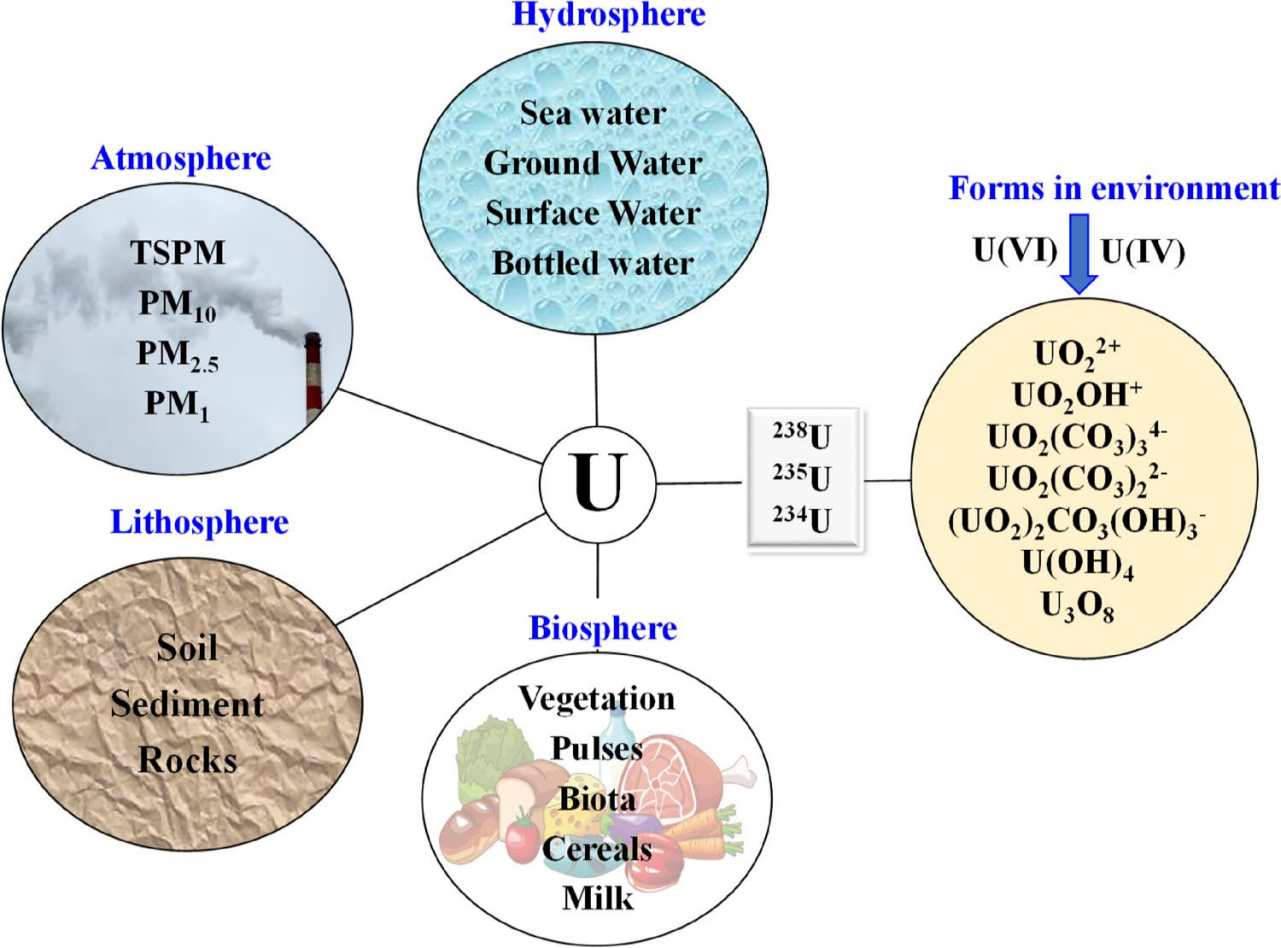
Distribution of uranium in the environment

The Earth's ecosystem is an intricate and interconnected dynamic system that encompasses diverse components such as rocks, soil, water, plants, and animals. This intricate network of interactions plays a vital role in sustaining life on our planet. Uranium, a naturally occurring element, has been present on Earth since its formation 4.5 billion years ago. Natural uranium consists of three isotopes: ^238^U (99.28%), ^235^U (0.70%), and ^234^U (0.0054%), distributed in both biotic and abiotic components of the environment. Geochemical and environmental factors have led to the dispersion of uranium from its minerals into the lithosphere, hydrosphere, biosphere, and atmosphere, as illustrated in (Fig. 1). The average uranium concentration in the Earth's crust is approximately 2.8 ppm. In ground waters and seawaters, uranium concentrations range from ≤ 0.001 to 0.008 ppm and nearly 3 µg/L, respectively (Balaram et al. 2022). Drinking water is a crucial element of the human diet and is considered in dietary intake estimates.

Despite being a radioactive element, uranium is also recognized for its chemical toxicity. When consumed, uranium primarily impacts the kidneys, potentially leading to kidney damage at excessively high concentrations. Notably, uranium toxicity predominantly affects the renal tubules (Kurttio et al. 2006). To safeguard human health from the potential adverse effects of uranium intake through the drinking water pathway, national and international advisory organizations/agencies, such as the International Commission on Radiological Protection (ICRP), the United States Environmental Protection Agency (USEPA), the World Health Organization (WHO), the Codex Alimentarius Commission (Codex), etc., establish guideline values or ensure their implementation through regulatory bodies. The frequent revisions in guideline values and discrepancies among different countries have heightened public concerns regarding the presence of uranium in drinking waters. It is important to note that the regulatory limits for uranium in drinking waters can vary between different countries due to several influencing factors. The driving force behind these inconsistencies include variations in the primary basis for formulating standards, encompassing the sensitivity of population groups, geological conditions, economic conditions, and more. In India, two different regulatory agencies, namely the Atomic Energy Regulatory Board (AERB) and the Bureau of Indian Standards (BIS), have stipulated distinct limits for uranium in drinking water. In the conventional approach based on dose limits, doses exceeding the limit are deemed intolerable, while doses below the limit are kept as low as reasonably achievable, a methodology differing from that employed in the case of exposure to hazardous chemicals. Generally, ensuring safe drinking water aims for a goal of zero risk to the public within technical and financial limitations.

The World Health Organization (WHO) releases manuals, handbooks, recommendations, and guidelines that address health-related concerns related to pollutants and are recognized as global advisories (WHO 2011). Since 1958, the organization has consistently issued specific guidelines for international drinking water standards, presenting limits for both chemical (in mg/l) and radiological (in Bq/l) pollutants. The critical consideration for uranium revolves around whether the existing guideline value is adequate to prevent chemical toxicity in the kidneys. This determination hinges on various factors, including the assumed threshold for chemical toxicity in the kidney, the appropriate selection of an uncertainty factor, and the relationship between uranium kidney burden and dose. In 1987, the Agency for Toxic Substances and Disease Registry (ATSDR) published toxicological profiles containing information on the toxicology and adverse health effects associated with uranium. The initial recognition of uranium as a chemical toxicant occurred in ATSDR (1999), with a revised version published in ATSDR (2013).

The scope of this paper is to compare the procedures adopted in deriving guideline values of uranium in drinking water. A glimpse of the nationwide survey of uranium in Indian drinking waters has also been presented. The paper also discusses the diversity in methodologies, uncertainties in the input parameters, absence of harmonization in country-specific guidelines, and various sources of information related to animal studies and human data. The counterproductive outcomes stemming from the adoption of WHO recommendations for uranium in drinking water, particularly concerning socio-economic considerations for India has also been emphasized.

## Approaches for guideline values

**Fig. 2:**
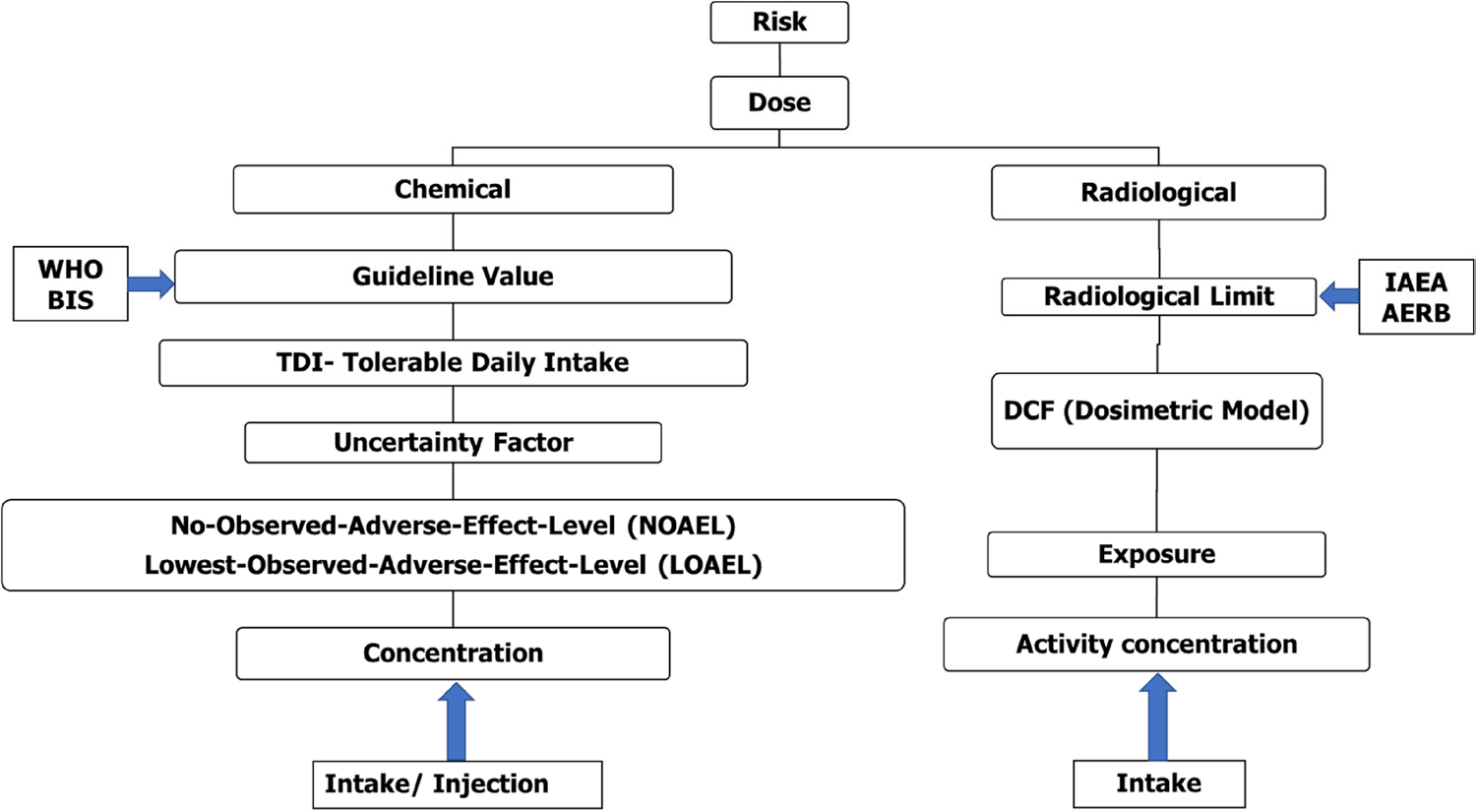
Methodology for derivation of chemical and radiological guideline value for Uranium

The Compendium of Chemical Terminology Gold Book by the International Union of Pure and Applied Chemistry (IUPAC 2014) presents two definitions of 'dose.' In toxicology, 'dose' refers to the entire amount of a chemical delivered to, ingested by, or absorbed by an organism, organ, or tissue, measured in mass units (µg, mg, or g). Conversely, in radiation and health physics, 'dose' signifies the amount of photon energy absorbed by an irradiated material per unit mass during a specific exposure period, expressed in J/kg or Gray (Gy) and referred to as absorbed dose. This absorbed dose is then adjusted for radiation and tissue weighing factors, converted into equivalent dose or effective dose, and expressed in Sievert (Sv) (ICRP 1990). These two concepts and their units are distinct, with the former relating to chemically toxic elements and the latter to radiologically toxic ones. However, uranium, as an element, possesses both chemical and radiological toxicity. Figure 2 illustrates the methodology employed to assess the risk posed by uranium as a chemical and radiological contaminant.

It is widely acknowledged that there exists a threshold dose below which the majority of potential chemical contaminants or toxicants do not induce adverse effects. For chemicals causing such toxic effects, a Tolerable Daily Intake (TDI) is established, as depicted in Fig. 2. The TDI is expressed on a body weight basis, for example, in mg/kg or μg/kg of body weight, as illustrated in Eq. 1.

1 $$\text{TDI}=\text{LOAEL or NOAEL }/\text{Uncertainty Factor}$$

where, NOAEL = no-observed-adverse-effect level,

LOAEL = lowest-observed-adverse-effect level,

UF = uncertainty factor

2 $$\text{Guideline value }= (\text{TDI }*\text{ BW }*\text{ P})/\text{ C}$$

where, BW = body weight (60 kg for adults, 10 kg for children, 5 kg for infants),

P = fraction of the TDI allocated to drinking-water,

C = daily drinking water consumption (2 L for adults, 1 L for children, 0.75 L for infants).

Guideline values founded on radiological considerations can be derived by following the limits recommended by the International Commission on Radiological Protection (ICRP). This global non-governmental organization comprises specialists from various domains, including radiation safety, medical physics, industrial hygiene, environmental protection, and emergency control. The ICRP regularly issues guidelines for the safety of occupational workers, the public, and the environment concerning radiological risk assessment and mitigation. The International Atomic Energy Agency (IAEA) and other professional organizations such as the World Health Organization (WHO), International Labour Organization (ILO), etc., endorse guidelines for radiation safety based on these guidelines. The IAEA Basic Safety Standards (IAEA BSS) provides recommendations for dose conversion factors (DCF) in cases of the intake of radioactive materials through ingestion and inhalation pathways, relying on the ICRP Publication-68 (ICRP 1994), as depicted in Fig. 2.

The IAEA Basic Safety Standards (IAEA, 1996) and IAEA GSR-Part 3 (2014) incorporates the dose coefficients outlined in various ICRP Publications (1990, 1994), which are being continuously updated. Utilizing these dose coefficients, the Derived Water Concentration (DWC) for natural uranium corresponding to a reference exposure/dose value is determined. The IAEA-BSS GSR Part 3 (2014), considers exposure due to ingestion of radionuclides in drinking water as an existing exposure situation, and provides the specific reference level. Based on this, it can be worked out that the annual effective dose to the representative person is unlikely to exceed a value of 1 mSv. Considering the radiological perspective, the reference level for uranium in drinking water is calculated to be 60 µg/l amounting to a dose of 0.1 mSv/y from the water route for members of the general public, depending on the solubility class of the uranium compound (fast solubility class).

**Fig. 3:**
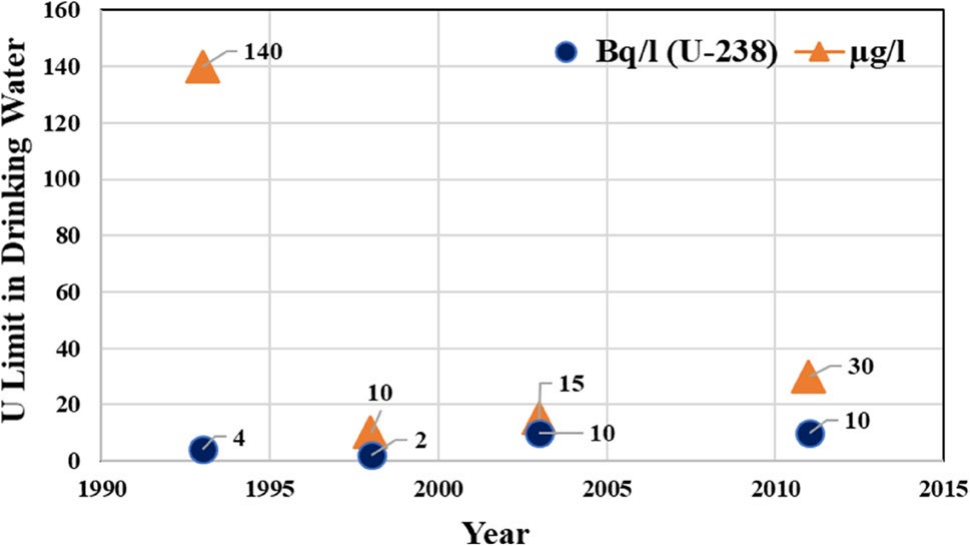
Evolution of the guideline value of uranium in drinking water by WHO

In the context of India, the derivation is based on the assumption of the average daily water intake of 4.05 L for an Indian citizen, and the lower limit for uranium in drinking water is set at 60 µg/l, presuming the most soluble radiological fast class (Sahoo et al. 2020 and references therein; AERB 2004; Raghavayya 1999). For ^238^U, based on a dose of 0.1mSv/a (Individual Dose Criteria—IDC), the guidance level equates to 3.04 Bq/l, which, when rounded logarithmically, would give the value of 10Bq/l. In the previous edition (WHO 2004) the guideline for ^238^U was set at 10 Bq/l (Fig. 3) and a provisional guideline at 15 µg/l, based on chemical toxicity. The latest WHO guidelines (2022) for drinking‑water quality, fourth edition, incorporates the first and second addenda retaining the guideline values based on radiological consideration for uranium isotopes for e.g. 10 Bq/l for ^238^U. The guidance levels were rounded to the nearest order of magnitude by averaging the log scale values thereby providing the guideline as 10 Bq /l for ^238^U. The guideline derived from the ingestion dose coefficient (ICRP 68) of 4.5 × 10^–8^ Sv/Bq, IDC of 0.1 mSv/y and 730 L/y has been worked out as 3 Bq/l for ^238^U. The guideline value of 10 Bq/l for ^238^U has been provided based on the consideration that the value 3 × 10^0^ be taken as 10^n+1^or 10^1^ Bq/l. The conservativism aspect of the application of the guideline values has been is discussed in WHO (2018).

In its initial release of the "Guidelines for Drinking Water Quality" (WHO 1984), the WHO determined that uranium did not necessitate any corrective measures. The second edition of the Guidelines for Drinking Water Quality, initially issued in 1993 and reissued in 1996, offered crucial insights to comprehend the underlying rationale for the guideline values. It's noteworthy that the (WHO 1993) Guideline for Drinking Water Quality recommends a guideline value of 140 µg/l for natural uranium, using 0.1 mSv/y as the benchmark value. The progression of the guideline values advocated by WHO is illustrated in Fig. 3.

The WHO introduces an initial guideline value of 2 µg/l in the 1998 addendum, primarily considering chemical toxicity. It is crucial to highlight that the document explicitly mentions the use of an uncertainty factor larger than 1000, and the provisional estimate relies on limited data regarding health effects. Additionally, the document underscores that the suggested guideline values are not legally binding limits. In this context, it is noteworthy that the 2004 guidelines from the WHO established a guideline value for uranium at 10 Bq/l and 15 µg/l. A subsequent revision in 2011 maintained the value of 10 Bq/l but introduced a new guideline value of 30 µg/l (Fig. 3). It's important to highlight that the current guideline value set by the United States Environmental Protection Agency (USEPA) for natural uranium is 30 µg/l, as indicated in 40 CFR (USEPA 2000).

## Health hazards of uranium

Uranium, as the heaviest naturally occurring alpha emitter, possesses an exceptionally low specific activity. Despite being a radioactive element, uranium lacks a known metabolic role in mammals and is therefore categorized as non-essential (Berlin & Rudell 1986). The primary routes of exposure to uranium are through the ingestion of food and beverages containing uranium isotopes and the inhalation of dust contaminated with uranium. When evaluating uranium toxicity, it is essential to consider both its chemical and radiological aspects. Unlike other radioactive elements, uranium concentrations in water samples are often specified similarly to those of chemical toxins, using units such as parts per billion (ppb or µg/l).

The majority of internally deposited uranium originates from gastrointestinal absorption following the intake of food or water. After ingestion, uranium is unable to reach the bloodstream and is almost entirely eliminated within a few days. Kidneys and bones serve as preferred sites for the deposition of small amounts of uranium (0.2 to 5%) that enter the bloodstream, with accumulations reaching around 12% and 22%, respectively. The remaining uranium is evenly distributed throughout the body and subsequently eliminated (WHO 2011). While the majority of uranium deposited in the kidneys exits within a few days, the portion deposited in bones can persist for many years. On average, the human body contains approximately 90 μg of uranium from normal intakes of water, food, and air.

### Animal studies

**Fig. 4:**
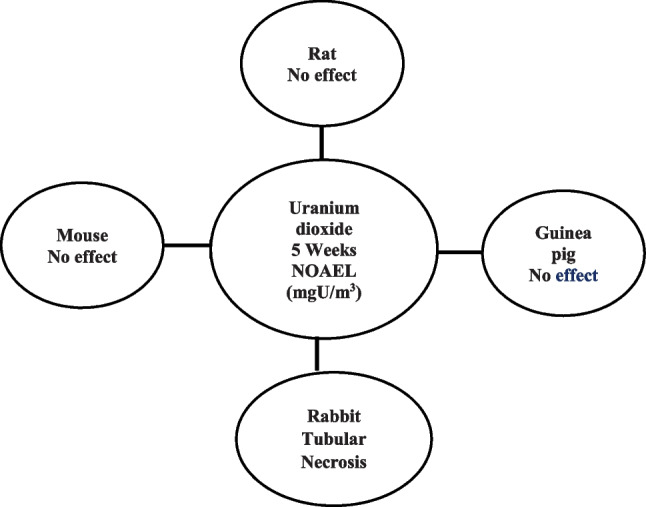
Effects on intermediate inhalation exposure to insoluble uranium compounds (ATSDR 2013)

Given the limited effects of uranium exposure in humans, much of our knowledge regarding uranium toxicity is derived from animal studies (Fig. 4). Numerous experiments on rats and mice were conducted worldwide, primarily in the 1940s and 1950s. The observed effects, especially in the proximal tubules, occurred when mice were exposed to uranium through inhalation, oral ingestion, or cutaneous routes (ATSDR 2013).

In a two-week research study led by Ortega and colleagues, forty male Sprague–Dawley rats were administered dosages of uranyl ethanoate dehydrate ranging from 0 to 16 mg/kg body weight in their drinking water. The rats exhibited a diverse array of metabolic effects (Ortega et al. 1989). In another experiment, conducted by Gilman et al. (1998a), fifteen male and fifteen female Sprague–Dawley rats were exposed to water containing uranyl nitrate hexahydrate at concentrations ranging from 0.001 to 600 mg/l for a duration of 91 days. Histopathological alterations were most prominent in the liver, thyroid, and kidneys.

In a parallel study, ten male New Zealand White rabbits were subjected to uranyl nitrate hexahydrate in their drinking water at doses ranging from 0.001 to 600 mg/l for 91 days, resulting in affected organs including the renal tubule, liver, thyroid, and aorta (Gilman et al. 1998b). Notably, histopathological abnormalities in the renal tubules were observed at concentrations above 0.96 mg/l of uranyl nitrate hexahydrate. Gilman et al. (1998c) conducted an additional investigation to assess the reversibility of renal damage in Pasteurella-free male New Zealand White rabbits. Over a 91-day period, groups of 5–8 animals were administered uranyl nitrate hexahydrate doses ranging from 0.001 to 600 mg/l, resulting in minor lesions in the liver, thyroid, and aorta.

Maynard & Hodge (1949) conducted research to investigate the effects of uranium ingestion on reproduction in animals. In a study involving rats, detrimental health effects were detected after seven months of dosing with 2% uranyl nitrate hexahydrate. In a separate study, Domingo et al. (1989a) administered doses of uranyl acetate dihydrate ranging from 0 to 50 mg/kg to groups of 20 pregnant Swiss mice to evaluate the developmental toxicity of uranium. The findings indicated a decrease in daily feed consumption, an increase in liver weight, and a reduction in the weight gain of the mothers. Fetal toxicity, diminished fetal weights and sizes, an increased number of impaired fetuses per litter, a higher incidence of both external and internal malformations, and an elevated prevalence of growth-related deviations were observed in mouse fetuses exposed to uranium doses of 2.8 mg/kg body weight per day and above.

Particular deformities, such as cleft palates and anomalies in development, were evident at uranium dosages of 14 mg/kg body weight per day and above. Notably, the uranium dosages used did not appear to be harmful to embryos. While uranium did not impact neuromotor maturity or development in young subjects, high doses of uranium were found to influence cognitive and memory capacities. The effects of intermediate inhalation exposure to insoluble uranium compounds are illustrated in Fig. 4.

In a subsequent study, Domingo et al. (1989b) assessed the effects of uranium on postnatal survival, gestation, slow fetal growth, and lactation. From day 13 of gestation to day 21 of nursing, groups of 20 female mice were subjected to varied dosages of uranyl acetate dihydrate ranging from 0 to 50 mg/kg body weight through gavage. Neither changes in dietary intake nor alterations in body weight indicated maternal poisoning.

The impact of uranium consumption on fertility rates in mice, along with pregnancy and postnatal survival, was investigated by Paternain et al. (1989). Before mating, followed by gestation, parturition, and nursing of young mice, adult male and female Swiss mice were administered oral uranyl acetate dihydrate dosages ranging from 0 to 25 mg/kg body weight daily for 60 days and 14 days, respectively. The observations revealed that mating and fertility were not adversely affected.

While high-specific-activity soluble uranium compounds have been demonstrated to induce bone cancer in experimental animals through injection or inhalation, no evidence of cancer has been found in animals consuming either insoluble or soluble uranium compounds (Wrenn et al. 1985).

### Epidemiological studies

Clinical investigations were carried out on 324 individuals in Nova Scotia, Canada, who were exposed to varying levels of natural uranium in drinking water, reaching up to 0.7 mg/l, sourced from private wells. No correlation was observed between uranium exposure and overt renal illness or other clinical complaints. However, there was a trend indicating an increased uranium concentration in well water associated with elevated urinary β-2-microglobulin excretion.

In Canada, the impact of uranium exposure through the drinking water pathway was studied on two distinct sets of subjects: one group exposed to < 1 µg/l and the other to 2–781 µg/l. According to Zamora et al. (1998), uranium concentrations had an impact on kidney function in the proximal tubule. In a 2002 study in Finland, individuals exposed to well water with a median uranium concentration of 28 µg/l were screened for potential kidney damage (Kurttio et al. 2006). While uranium in drinking water was statistically linked only to the excretion of calcium, a strong correlation was observed with the excretion of phosphate, glucose, and calcium in urine. The data suggested modest alterations in proximal tubular function, with no indication of any effect on glomerular function. No clear threshold to the observed effects was evident, but the study population was relatively small, and significant variation was observed within an unexposed population. The authors concluded that the clinical significance of the results could not be established.

The WHO recently concluded that there is no evidence of a correlation between the natural levels of uranium in drinking water and a carcinogenic effect (WHO 2011). While there was no suggestion of an impact on glomerular function, the results correlated with mild changes in proximal tubule function. The study sample was relatively small, and there was evidence of significant heterogeneity within an unexposed population. However, no indication of a clear threshold to the identified effects was found, making it challenging to determine the medical relevance of the outcome. Furthermore, the WHO emphasized that there is no proof associating uranium in drinking water with a cancer risk (WHO 2011).

Limited information is available on the toxicity of uranium to the kidneys of humans after inhalation exposure. Epidemiological studies on uranium industry workers found no change in mortality from kidney illness, and long-term exposure to low uranium levels did not appear to cause cases of kidney damage. However, increasing exposure to uranium was associated with renal impairment (β-2-microglobinuria, aminoaciduria). These studies indicated a potential correlation between the amount of uranium in potable water and general renal dysfunction parameters such as albumin, β2-microglobulin, glucose, and protein levels in the urine, but the biomarkers still fluctuated within the normal physiological range.

Therefore, while human oral exposure studies do not provide definitive dose–response data, they highlight the cytotoxicity of uranium. Studies conducted by Kurttio et al. (2006), considered by WHO, conclude that continuous uranium intake from drinking water, even at relatively high exposures, did not have cytotoxic effects on kidneys in humans. High doses of uranium have the potential to impair renal function, but these functional differences are less significant and highly uncertain from a clinical standpoint as they fall within the normal range. The link between uranium exposure and cancers of the lymphatic and hematopoietic tissues is not supported by significant research on the subject.

## Global scenario on limit of uranium in drinking water

The global landscape regarding the permissible limit of uranium in drinking water varies across different regions and countries. Diverse approaches and standards are employed by international and national regulatory bodies, leading to a lack of uniformity in the established guidelines. Various factors, including geological conditions, socio-economic considerations, and population dynamics, influence the decision-making process in determining these limits.

The World Health Organization (WHO) is one of the entities that periodically revises its guidelines for uranium in drinking water. These guidelines consider both chemical and radiological toxicity aspects. However, discrepancies in the recommendations exist among different countries and regions, leading to public concern and apprehension.

In some cases, national regulatory agencies set their own limits for uranium in drinking water, taking into account local factors and conditions. This decentralized approach acknowledges the need for country-specific standards, recognizing the influence of factors such as economic conditions, geological characteristics, and the sensitivity of different population groups.

The evolving nature of these guidelines, along with uncertainties associated with input parameters and a lack of harmonization in country-specific standards, underscores the complexity of establishing a global consensus on the acceptable limit of uranium in drinking water. The global scenario reflects the ongoing challenges in achieving a standardized and universally accepted approach to addressing the presence of uranium in this vital resource.

**Fig. 5:**
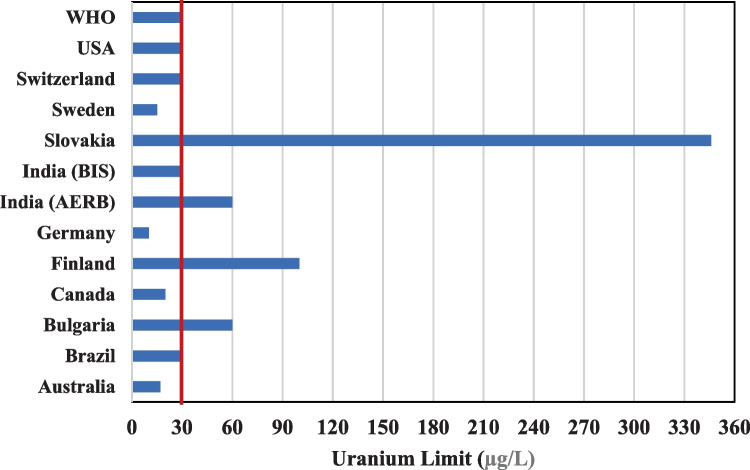
Global limits/ guideline values for uranium in drinking waters

In its water quality guidelines, the World Health Organization (WHO) explicitly states that individual countries have the discretion to establish distinct national guideline values, taking into consideration specific parameters such as local geological conditions, socio-economic factors, the prevalence of uranium in the environment, and potential health risks to the population. Some country-specific guidelines deviate from the WHO's guideline value of 30 µg/l, with values either exceeding or falling below this threshold. Additionally, countries may adopt either radiological or chemical approaches in formulating their guideline values, as illustrated in Fig. 5.

The literature underscores a high degree of uncertainty in toxicological and health data related to uranium exposure (WHO 2011; UNSCEAR 2016a, b). Regulatory agencies conduct risk assessments to establish safe levels of uranium in drinking water. International regulatory bodies periodically revise their recommendations for guideline values based on scientific research encompassing health-based studies, clinical findings, animal studies, and epidemiological investigations (WHO 1993, 2002, 2011, and 2012).

The United States Environmental Protection Agency (USEPA) employs the Linear No-Threshold (LNT) model for assessing the risk associated with uranium and derives the health-based Maximum Contamination Level Goal (MCGL). Utilizing the LOAEL values and appropriate uncertainty factors (UFs), the Reference Dose (RfD) is proposed, considering health-based and epidemiological data. The RfD of 0.6 μg/kg/day is derived from the applicable LOAEL and UFs. Although the Drinking Water Equivalent Levels (DWEL) for uranium were initially set at 20 μg/l, the USEPA took into account technical limitations, scientific observations, and a cost–benefit analysis, ultimately adopting a Maximum Contamination Level (MCL) of 30 μg/l (USEPA 2000).

In formulating a country-specific limit, the Canadian agency categorized uranium as a chemically toxic element. Considering standard parameters of body weight, a drinking water intake of 1.5 L, and assigning 50% of the Tolerable Daily Intake (TDI) to the drinking water pathway, the country derived a health-based value of 14 μg/l. Since no additional health benefits were expected, the guideline value of 20 μg/l was ultimately adopted (Health Canada 2019).

The Australian agency considered both the radiological and chemical perspectives in arriving at the country-specific guideline value. Adopting an approach similar to the WHO but differing in the considered body weight (70 kg), they arrived at a limit of 17 μg/l based on the chemical toxicity of uranium. From the radiological perspective, a value of 3 Bq/l was derived, corresponding to 240 μg/l (ADWG, 2011). However, the final recommended limit was 17 μg/l.

Sweden incorporated a pivotal animal experiment and three epidemiological studies to establish a country-specific intervention level of 15 μg/l for uranium in drinking water (Svensson et al. 2005). The authors recommended that regulatory bodies consider epidemiological data when recommending guideline values or limits.

Germany, akin to the WHO, considered uranium as a chemically toxic element but applied an increased safety factor of 3, setting 10 μg/l as the national binding guideline value (Banning and Belfer 2017).

## Uranium in Indian drinking waters at a glance

Various localized studies for uranium in drinking water have been conducted in different regions of India, reporting elevated levels in certain areas. In a vast and diverse country like India, relying on limited samples from fixed sources, methodological variations, procedural limitations and a lack of harmonized approach is unlikely to accurately reflect the prevailing environmental conditions. Numerous sporadic and localized studies have reported increased levels of uranium in groundwater, particularly in regions like Punjab, Chickballapur in Karnataka, and the Lalbara region of Madhya Pradesh (Babu et al. 2008; Singh et al. 1995; Sinha et al. 1997). Case studies from the Malwa region of Punjab, where elevated uranium levels were reported in groundwater, led to the initiation of a comprehensive survey programme.

The Health Physics Division of Bhabha Atomic Research Centre has established a nationwide database for uranium in drinking waters using an organized and graded methodology following international practices. This database confirms the presence of detectable uranium concentrations across regions with diverse geological, cultural, and socio-economic conditions. In addition to determining uranium levels in drinking water, the assessment included calculating ingestion dose to the public through drinking water exposure pathway. The study measured uranium concentrations, sixteen water quality parameters, GPS details, gamma dose rate, type of water source, water table depth, and surrounding rock type. Further details of the programme can be found elsewhere (Sahoo et al. 2020).

### Observations from nationwide survey

**Fig. 6:**
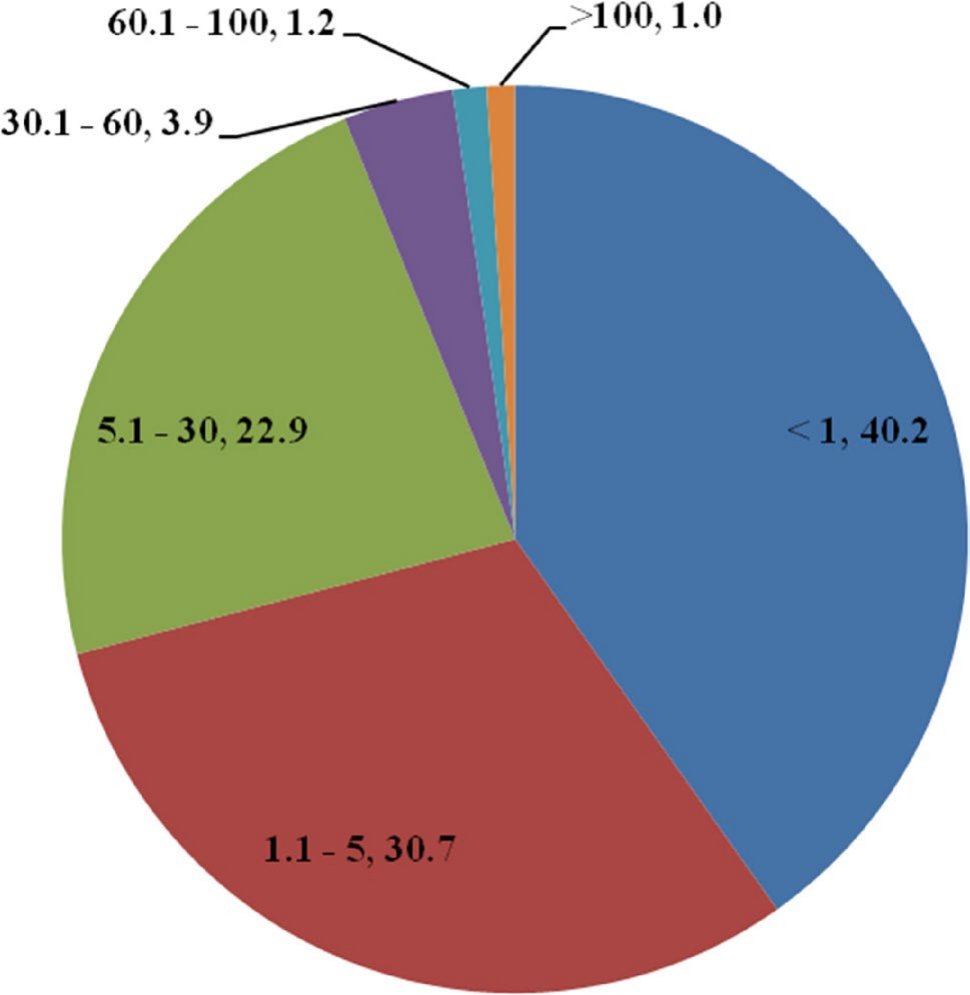
Frequency distribution of uranium content (µg/L) in groundwater

A nationwide survey conducted on various drinking water sources throughout India revealed that uranium concentrations in surface water are considerably lower than those in groundwater. An optimised grid size of 36 km^2^ was used. Out of 718 districts of the country, 403 districts in 23 States and 3 Union Territories are covered. Uranium and other water quality parameters have been measured in fifty-five thousand five hundred and fifty-four (55,554) samples. Geographical attributes, uranium and sixteen associated water quality parameters in drinking water as well as ambient gamma radiation level of the adjacent area have been reported. Uranium concentration in water samples ranged from 0.2—4918 µg/L (µg/l). Uranium content in 98% of the samples was found to be less than 60 µg/L set by AERB, India. The pie-chart of the data is given in Fig. 6. From the figure, it can be observed that 93.9% and 97.8% of the data lie below the WHO guideline value of 30 µg/L and the AERB limit of 60 µg/L, respectively. It is observed that 2.2% of the samples had uranium concentrations > 60 µg/L in both pre-monsoon and post-monsoon seasons. Only 1% samples had uranium concentrations above 100 µg/L.

**Fig. 7:**
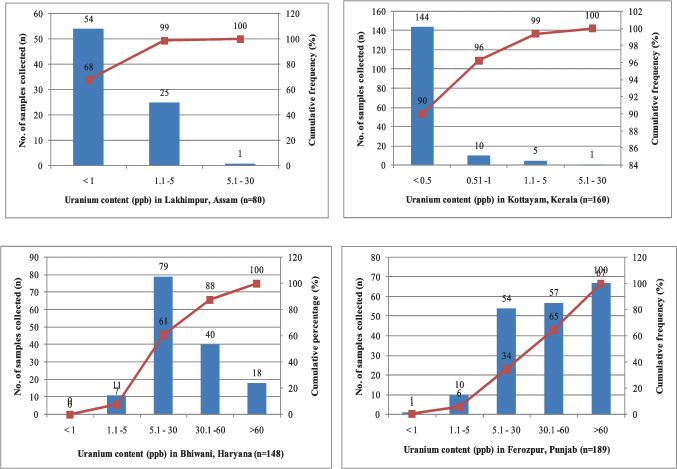
Variation of U content in water samples in different aquifers

**Fig. 8:**
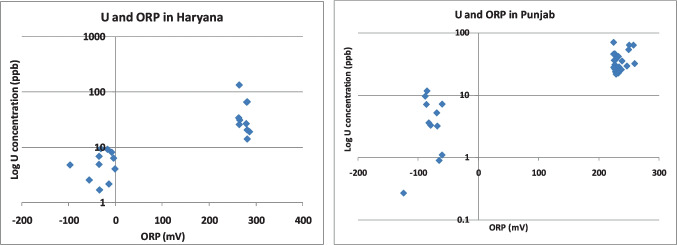
Variation of U concentration in groundwater with redox potential

Lower levels of uranium have been noted in shallow aquifers located in coastal regions, primarily in areas with high rainfall. In contrast, higher concentrations are observed in deeper aquifers in regions with moderate rainfall (Fig. 7). Aquifers exhibiting oxidizing conditions tend to contain higher uranium concentrations (Fig. 8), while deep aquifers generally exhibit lower uranium concentrations. Unusually high concentrations of uranium in groundwater are observed in aquifers that are typically deeper and face challenges in receiving sufficient recharge due to associated geomorphology. In certain cases, other contributing factors such as inorganic complexation, the presence of humic compounds, and regional geochemistry may play a significant role. The distribution of uranium in groundwater and an aquifer's susceptibility to high uranium content can be influenced by multiple factors, and these factors may not be isolated or linked to a single cause. Studies in India have concluded that in 97.8% of the samples, uranium contents fall below the recommended drinking water limit set by the AERB (Jha et al. 2021).

## India specific Guideline Values

Based on the recommendations issued by the ICRP, as periodically amended, the IAEA establishes primary limits for occupational and public radiation exposure. These primary limits are enforced by the AERB, which serves as India's national regulatory body for ionizing radiation. In fulfilling its regulatory mandate for nuclear facilities and the handling of radioactive sources with minimal impact on occupational workers, the public, and the environment, the AERB provides guidelines and stipulations through various publications.

**Fig. 9:**
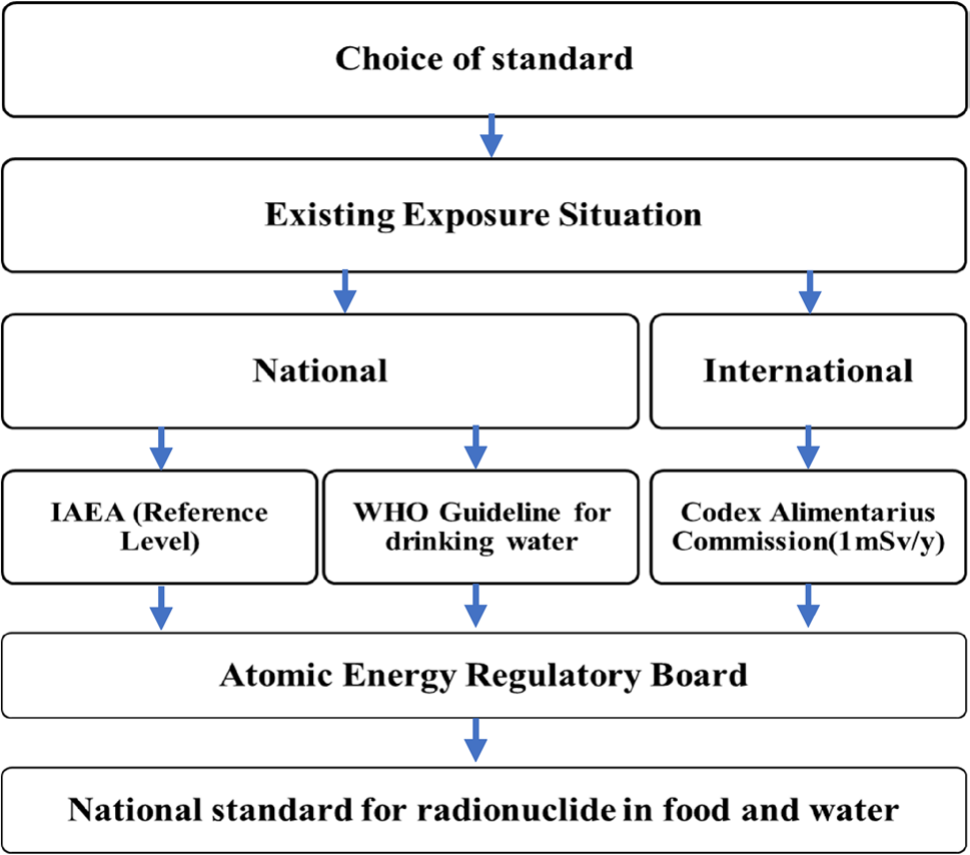
Adoption of guidelines by National regulator (radiological) in India

The current exposure limits resulting from practices, beyond ambient background radiation levels, are set at 20 mSv/y for occupational workers and 1 mSv/y for members of the public. Secondary limits for various exposure pathways of relevant radionuclides, based on these primary limits, are determined using the Annual Limits on Intake (ALI) or Dose Conversion Factors (DCFs) provided by the ICRP or the IAEA Basic Safety Standards (BSS). The AERB follows the procedure outlined in Fig. 9 to establish country-specific limits. When formulating national limits, emphasis is placed on technical justifications that take into account India's socioeconomic conditions without compromising the underlying radiological safety directives. The agency has prescribed a drinking water limit for uranium based on radiological toxicity (Sahoo et al. 2020 and references therein; AERB 2004).

The methodology employed by the Health Physics Division of Bhabha Atomic Research Centre, India, before 2004, involves the allocation of the primary dose limit of 1 mSv, with contributions as follows: i) 50% through water consumption, ii) 30% from air inhalation, and iii) 20% from all other routes. This approach is utilized to determine the secondary limits for natural uranium in water. The proportion of the ingestion dose attributable to uranium is calculated based on exposure through 1/5th of the water route, amounting to 0.1 mSv/y (Raghavayya 1999). By assigning a value of 0.1 mSv/y for the dose arising from uranium ingestion via water, the derived water concentration (DWC) is computed using the Annual Limit on Intake (ALI) value provided in ICRP-68 (ICRP 1994). Consequently, the limit for natural uranium in drinking water is considered to be the most stringent value of 100 µg/l within the Health Physics community of India (Sahoo et al. 2020). The derived limits for natural uranium in drinking water for members of the public, calculated based on a reference exposure of 0.1 mSv/y from relevant ICRP and IAEA documents, are compiled in Table 1. Additionally, WHO (2022) provides rationale for the screening levels and guidance level which accounts both the existing and planned exposure situations.

**Table 1:** Derived limit for natural uranium in drinking water for members of public

Sl. No	Source document	Derived water concentration of uranium		
		Radiological	Chemical	Remarks
		Bql^−1^	µgl^−1^	
1	WHO – Guidelines for drinking water quality, 2nd Edition, Vol.- 1 (WHO 1993)	-	-	Reference value Gross alpha: 0.1 Bql^−1^ Gross beta: 1.0 Bql^−1^
	(a) By mass		140	-
	(b) By activity	4	160	-
	(c) Addendum (WHO 1998)	-	2	Provisional value based on limited information on health effects or using uncertainty factor > 1000, emphasizing in the introduction that these are not mandatory limit
2	4th Edition (WHO 2022)	10^*^	-	-
3	Indian Bureau of Standards BIS (1991)	-	-	Gross alpha: 0.1 Bql^−1^ Gross beta: 1.0 Bql^−1^
4	Indian Bureau of Standards BIS (2021)	-	30	-
5	US-EPA (2000)	-	30	Effective Dec. 8, 2003. Applicable for drinking water supplies

* U-238

The Indian Standard IS 10500:2012 for Drinking Water specifications has established maximum acceptable limits for radioactive residues, specifically alpha and beta emitters (BIS 2012). Exceeding these specified values renders the water unsuitable for consumption. These requirements encompass all radioactive elements, including uranium, without singling out individual radionuclides. The Bureau of Indian Standards (BIS) recommends a two-tier approach, where drinking water undergoes screening for gross activities of alpha and beta emitters. If the predetermined activity levels of 0.5 Bq/l (for alpha) and 1 Bq/l (for beta) are surpassed, further investigation into individual radionuclides becomes necessary. Recently, the Bureau of Indian Standards (BIS 2021) has adopted a national limit of 30 µg/l, aligning with the WHO guideline value. It is noteworthy that in adopting these guidelines, BIS did not conduct health-based investigations specific to the Indian population, nor did it consider experimental and epidemiological studies, scientific and technical bases, or engage in remediation and cost–benefit analysis. Importantly, the Atomic Energy Regulatory Board (AERB), serving as the national regulator for radiological purposes, has established a national limit of 60 µg/l based on the best scientific judgments (Table 1). This limit may be deemed reasonable due to the following factors: a) the cost associated with reducing the concentration to extremely low levels cannot be justified by the corresponding benefits, and b) it is consistent with values prescribed by many other countries and aligns with recommendations from international organizations.

### Challenges in adopting WHO guideline value in India

The strategy for addressing elevated levels of uranium, greater than the stipulated drinking water limit of 60 µg/l, as per AERB, or 30 µg/l, as per WHO guideline, involves either transitioning to alternative, uranium-free water sources like surface water or implementing uranium removal from groundwater when alternative water sources are unavailable.

In areas with high uranium concentrations in groundwater, efforts are typically directed towards immediately shifting the water supply source to the nearest surface water or blending groundwater with surface water at the municipal supply point. Blending entails diluting contaminated groundwater with another water source containing low or minimal uranium content, effectively reducing uranium concentrations to within permissible limits or guideline values.

The other technologies for removal of uranium from drinking water are: (i) coagulation/filtration, (ii) anion exchange, and (iii) reverse osmosis (RO) (Katsoyiannis and Zouboulis 2013). The selection of an appropriate treatment method for uranium removal requires a comprehensive understanding of the uranium species present in water, which can be identified using water quality parameters such as pH, total dissolved solids (TDS), alkalinity, calcium, and total organic carbon (TOC). Each of the techniques has certain limitations but Ion exchange /RO technique has been effectively utilized at many locations with elevated levels of uranium in drinking water.

Coagulation/filtration, a prevalent water treatment process, removes uranium through adsorption and coprecipitation of the dissolved uranyl complexes by coagulant precipitates. For the community, high-capacity systems are required for ensuring the availability of potable water to a large population. Along with the basic features of a water treatment plant specific requirements can be met using various technologies such as ion exchange, reverse osmosis and ultra-filtration etc. or a combination of technologies. The RO based systems have a significant drawback in generating a 'reject' stream with elevated uranium concentrations, necessitating proper management of secondary liquid waste to prevent environmental contamination. Hence generation of secondary waste may pose an additional burden on the society while supplying potable water to the public.

Due to ease of handling, maintenance and associated technological advantages RO based systems are normally preferred over other technologies. Water with elevated uranium concentration is passed through the RO based system and potable water is obtained. A RO plant with a capacity of 500 LPH and operating for 6 h/d generates 3000 l/d potable water. Assuming an individual uses 10 L water per day, a population of 100,000 would require 1 million liters of water. This would require setting up of nearly 334 RO plants to cater to a population of 100,000. Considering an investment of 500,000 Indian rupees (INR) per plant, this would require INR 170 million to cater to the potable water requirements of 100,000 people. This excludes the cost of land acquired and revenue required for safe disposal and long-term monitoring of the residue. Considering an ion-exchange plant with similar parameters as the RO plant mentioned earlier. To supply potable water to a population of 100,000 people, nearly 334 ion-exchange plants have to be set up. Considering an investment of INR 800,000 per plant, this would require INR 270 million to cater to the potable water requirements of 100,000 people. This excludes the cost of land acquired and revenue required for safe disposal and long-term monitoring of the residue. Since the number of people affected is directly related to the drinking water limit, the investment may increase manifold.

Due to poor desorption/removal efficiency, the lean media won’t qualify as exempt waste. Settling sludge (prior to ion exchange feed), exhausted resin, filter bed material and other sorbents normally form the inventory of solid waste. Rinse and backwash, regenerate solution, reject water and in certain cases feed water can form part of the liquid waste. Assuming an affected population of 300,000 and considering BIS-prescribed permissible limit of uranium in drinking water as 30 µg/l, with a feed containing 70 µg/l of uranium, 3 million litres of water need to be purified for drinking purposes. This will lead to the generation of 150 g uranium per day on ion exchange material (amounting to 54.75 kg of uranium per year). Apart from the known hazardous issues, the radiological considerations are also important for the stakeholders (workers, operators and public). This may require monitoring around waste disposal facilities for containing any environmental leaching from the generated waste. Additionally, the waste management practices for a long period of time would pose technological challenges and incur additional costs.

When selecting a treatment technology, careful assessment of uranium and co-contaminant concentrations in waste residue, sludge, and backwash is essential to ensure that disposal options comply with regulatory requirements and are technically and economically viable. The USEPA addressed these considerations when establishing the country-specific Maximum Contamination Level (MCL) value for uranium in drinking water. The EPA's analysis demonstrated that there is no predictable difference in health effects and kidney toxicity risks between MCLs of 20 µg/l, 30 µg/l, and 80 µg/l. The cost–benefit analysis included incremental annual cancer risk reductions, total national annual compliance costs, and monetized benefits, excluding kidney toxicity. The conclusion was that the incremental benefits of implementing an MCL of 30 µg/l are significantly greater than those for an MCL of 20 µg/l. Consequently, the EPA established 30 µg/l as the drinking water limit for uranium to protect the general population, including children and the elderly (USEPA 2000).

## Uncertainty in assigning guideline values

The health studies conducted have not provided clear NOAEL (No-Observed-Adverse-Effect-Level) and LOAEL (Lowest-Observed-Adverse-Effect-Level) values, leading to a considerable degree of uncertainty associated with the guideline values established by regulatory authorities. Uncertainty in analytical results encompasses errors and variations inherent in the measurement process, including random uncertainty (precision), systematic uncertainty (accuracy), instrument drift, uncertainty in calibration standards, variation in samples, matrix effects, interference, chemical speciation, and environmental conditions. Uncertainty in uranium concentrations at the source and point of human intake, especially in potable waters, can significantly impact the quality and reliability of environmental data used for epidemiological studies.

Uncertainty arising from health study data is a complex issue, necessitating consideration of various sources, such as measurement errors in estimating uranium exposure levels, timing, duration, magnitude of exposure, classification of individuals into different exposure groups, and the presence of confounding factors like tobacco use, occupational exposures, alcohol consumption, and exposure to other toxicants. Extrapolating health study outcomes from higher to lower levels introduces a high degree of uncertainty. The lack of representative data on uranium levels in food and associated uncertainty poses challenges when assessing potential health risks through dietary intake. Additionally, uncertainty in water consumption data due to variations across countries, regions, population groups, and individuals can hinder accurate assessments of intake and exposure.

In the context of environmental and occupational exposure scenarios for uranium, conservatism refers to a cautious approach when assessing and managing potential risks. Conservative exposure scenarios prioritize protecting vulnerable populations, such as children, pregnant women, senior citizens, and individuals with pre-existing health conditions. Striking a balance between health protection and enabling productive activities requires careful consideration of dose/risk and expert judgment. As more research becomes available, exposure scenarios and risk assessments may be updated for a more accurate understanding of potential risks associated with uranium in drinking water.

Various methodologies for treating water with high uranium concentrations, such as filtration, ion exchange, reverse osmosis, precipitation, nanofiltration, and softening by lime adsorption on active charcoal and zerovalent iron, are practiced globally. However, the implementation of purification technologies comes with financial liabilities, and national standards should align with the perceived risks and provide greater net benefits to the concerned society. Residues from purification systems and rejects may also pose environmental concerns. While conservatism is crucial for ensuring safety in situations with scientific uncertainties, it should not hinder economic or societal progress unnecessarily, creating an additional burden in providing uranium-free drinking water to the population.

Considering the factors contributing to uncertainty in exposure assessment due to uranium, different countries have established varying limits and guideline values for uranium in potable waters, ranging from 2–964 µg/l, as indicated in Table 1. It is noteworthy that the WHO recommends the adoption of national guideline values, considering several country-specific factors, rather than merely adopting the WHO's guideline value of 30 µg/l.

## Conclusions

There exists a notable disparity in the limits and guideline values provided for uranium in drinking water among various countries worldwide and even within national agencies. Significant variations between the guidelines issued by the WHO and individual organizations are evident in numerous instances. The derivation of national limits should take care of multiple variables, such as geological features, technological challenges, waste management practices, environmental attributes, economic feasibility, socio-economic conditions, and confounding factors. The AERB limit of 60 µg/l was prevailing for last two decades. The BIS limit of 30 µg/l should result in net societal benefit. It is pertinent to mention that before enforcing a national limit it is a prerequisite to conduct health based and epidemiological studies in the country. The existing scientific knowledge about the health effects of uranium, especially at lower concentrations, may have large uncertainties. Since multiple issues influence the limit of uranium in drinking water, it would be prudent to continue with the AERB limit of 60 µg/l.
