# Correction to: Uranium standards in drinking water: An examination from scientific and socio‑economic standpoints of India

**DOI:** 10.1007/s11356-024-35782-6

**Published:** 2024-12-27

**Authors:** Sanjay K. Jha, Aditi C. Patra, Gopal P. Verma, Vivekanand Jha, Dinesh K. Aswal

**Affiliations:** 1 Health Physics Division, Health Safety & Environment Group, BARC, Mumbai, 400085 India; 2 Health Safety & Environment Group, BARC, Mumbai, 400085 India; 3 Homi Bhabha National Institute, Anushaktinagar, Mumbai, 400094 India

**Correction to: Environmental Science and Pollution Research (2024) 31:47461–47474**

10.1007/s11356-024-34352-0

The authors wish to issue an erratum regarding an inadvertent error in a reference in the aforementioned paper. Specifically, in the reference section on page no. 47473, the 20th reference was listed incorrectly. Dr. S. K. Jha is the corresponding author of the reference in question. The correct reference is:

Existing reference: Jha SK, Sahoo SK, Jha VN, Patra AC, Kulkarni MS (2021) Survey of Uranium in drinking water sources in India: Interim Observations. Curr Sci 120(9):1482–1490. 10.18520/cs/v120/i9/1482-1490.

Correct reference: Sahoo SK, Jha SK, Jha VN, Patra AC, Kulkarni MS (2021) Survey of Uranium in drinking water sources in India: Interim Observations. Curr Sci 120(9):1482–1490. 10.18520/cs/v120/i9/1482-1490.

